# Platelet factor 4 limits neutrophil extracellular trap– and cell-free DNA–induced thrombogenicity and endothelial injury

**DOI:** 10.1172/jci.insight.171054

**Published:** 2023-11-22

**Authors:** Anh T.P. Ngo, Abigail Skidmore, Jenna Oberg, Irene Yarovoi, Amrita Sarkar, Nate Levine, Veronica Bochenek, Guohua Zhao, Lubica Rauova, M. Anna Kowalska, Kaitlyn Eckart, Nilam S. Mangalmurti, Ann Rux, Douglas B. Cines, Mortimer Poncz, Kandace Gollomp

**Affiliations:** 1 Division of Hematology, Children’s Hospital of Philadelphia, Philadelphia, Pennsylvania, USA.; 2 Department of Pediatrics, Perelman School of Medicine at the University of Pennsylvania, Philadelphia, Pennsylvania, USA.; 3 Institute of Medical Biology, Polish Academy of Science, Lodz, Poland.; 4 Department of Medicine, and; 5 Department of Pathology and Laboratory Medicine, Perelman School of Medicine at the University of Pennsylvania, Philadelphia, Pennsylvania, USA.

**Keywords:** Hematology, Inflammation, Neutrophils, Platelets, Thrombosis

## Abstract

Plasma cell-free DNA (cfDNA), a marker of disease severity in sepsis, is a recognized driver of thromboinflammation and a potential therapeutic target. In sepsis, plasma cfDNA is mostly derived from neutrophil extracellular trap (NET) degradation. Proposed NET-directed therapeutic strategies include preventing NET formation or accelerating NET degradation. However, NET digestion liberates pathogens and releases cfDNA that promote thrombosis and endothelial cell injury. We propose an alternative strategy of cfDNA and NET stabilization with chemokine platelet factor 4 (PF4, CXCL4). We previously showed that human PF4 (hPF4) enhances NET-mediated microbial entrapment. We now show that hPF4 interferes with thrombogenicity of cfDNA and NETs by preventing their cleavage to short-fragment and single-stranded cfDNA that more effectively activates the contact pathway of coagulation. In vitro, hPF4 also inhibits cfDNA-induced endothelial tissue factor surface expression and von Willebrand factor release. In vivo, hPF4 expression reduced plasma thrombin-antithrombin (TAT) levels in animals infused with exogenous cfDNA. Following lipopolysaccharide challenge, *Cxcl4^–/–^* mice had significant elevation in plasma TAT, cfDNA, and cystatin C levels, effects prevented by hPF4 infusion. These results show that hPF4 interacts with cfDNA and NETs to limit thrombosis and endothelial injury, an observation of potential clinical benefit in the treatment of sepsis.

## Introduction

Sepsis is a dysfunctional response to infection that leads to life-threatening organ damage. Although sepsis is a leading cause of mortality worldwide, treatment remains limited to antibiotics and supportive care (1, 2). Over the past decade, multiple studies have shown that levels of plasma cell-free DNA (cfDNA) are highly elevated in septic patient plasma and are associated with organ dysfunction and mortality (3–6). Recent studies have shown that cfDNA is not merely a marker of disease severity, but actively contributes to disease progression by activating the intrinsic pathway of coagulation and acting as a damage-associated molecular pattern (DAMP) (7). In sepsis, elevated cfDNA originates from necrosis, apoptosis, pyroptosis, and increased red blood cell turnover, but in many patients, the main source is neutrophil extracellular traps (NETs) (8), webs of negatively charged, genomic DNA complexed with positively charged histones and neutrophil-released antimicrobial proteins that capture and kill pathogens (9–11). When NETs are degraded by circulating DNases, they release NET degradation products (NDPs), including cfDNA, histones, and neutrophil granule proteins that trigger prothrombotic pathways and cause oxidative vascular damage (12–16).

Based on the finding that NDP levels correlate with end-organ damage and mortality in septic patients (3, 5, 17–20), it has been proposed that preventing NET formation might be beneficial (21, 22); however, NDP levels are already elevated in septic patients at clinical presentation (17, 23–25), and blocking NET release on admission may be too late to affect outcome. Another proposed NET-directed strategy is to degrade NETs by infusing DNases (26–29); however, DNase I does not remove NDPs such as histones from the vascular wall (30), and studies of sepsis in murine models suggest that the infusion of DNase I early in the course of infection liberates entrapped bacteria, increases levels of circulating NDPs, and triggers the release of inflammatory cytokines, leading to worse outcomes (31, 32).

We have previously proposed an alternative NET-based strategy to treat sepsis. Platelet factor 4 (PF4, CXCL4) is a chemokine stored in platelet α granules that constitutes 2%–3% of total platelet releasate, and local concentrations exceed 12 μg/mL at sites of vascular injury (33). Due to its strong positive charge, PF4 tetramers aggregate polyanions such as heparin (34) and NETs (13, 32). Prior studies by our group demonstrated that human PF4 (hPF4) improves survival in a murine lipopolysaccharide (LPS) endotoxemia model of sepsis (35). We showed that hPF4 exerts this protective effect, in part, by binding to NETs, causing them to become physically compact, while enhancing their resistance to nuclease digestion (32). KKO, a monoclonal antibody directed against hPF4-polyanion complexes (36), binds hPF4-NET complexes, further enhancing their resistance to nuclease digestion. A deglycosylated version of KKO, which works in concert with hPF4 to reduce NDP release and enhance NET capture of bacteria, markedly improved survival in murine LPS endotoxemia and polymicrobial sepsis (13, 32, 37).

It may seem counterintuitive to stabilize NETs in sepsis, as they are considered to be thrombogenic and injurious to the endothelial lining (12–15, 17). Genomic sequencing efforts revealed that plasma cfDNA in septic patients is highly digested, with fragment sizes ranging from 150 base pairs (bp) to 200 bp, with a higher concentration of short-fragment cfDNA in patients with sepsis (5, 38, 39). Here, we compared the thrombogenic effects of high-molecular-weight (HMW) genomic DNA and intact HMW NETs to low-molecular-weight (LMW) DNA and highly digested LMW NETs. Our results are consistent with prior studies that show intact NETs composed of DNA complexed with histones have limited in vitro thrombin generating potential (40). We extend these findings to show that HMW, but not LMW, DNA and NETs have limited thrombogenicity in thrombin and fibrin generation assays. This difference likely involves the presence of greater levels of single-stranded DNA (ssDNA) in LMW DNA and NETs. The addition of hPF4 at physiologic concentrations reduces the thrombogenicity of LMW NETs, LMW DNA, and of ssDNA. We also examined the effects of NETs and DNA of variable length on vascular injury, and found that while incubation with HMW DNA and NETs did not change endothelial behavior, incubation with LMW DNA and NETs promotes endothelial release of von Willebrand factor (VWF) and tissue factor (TF) expression via activation of Toll-like receptor 9 (TLR9). Corollary studies in mice demonstrated that hPF4 reduces in vivo thrombosis and organ damage associated with elevated plasma cfDNA and endotoxemia.

## Results

### More rapid fibrin generation by LMW than HMW NETs and cfDNA.

**Figure 1:**
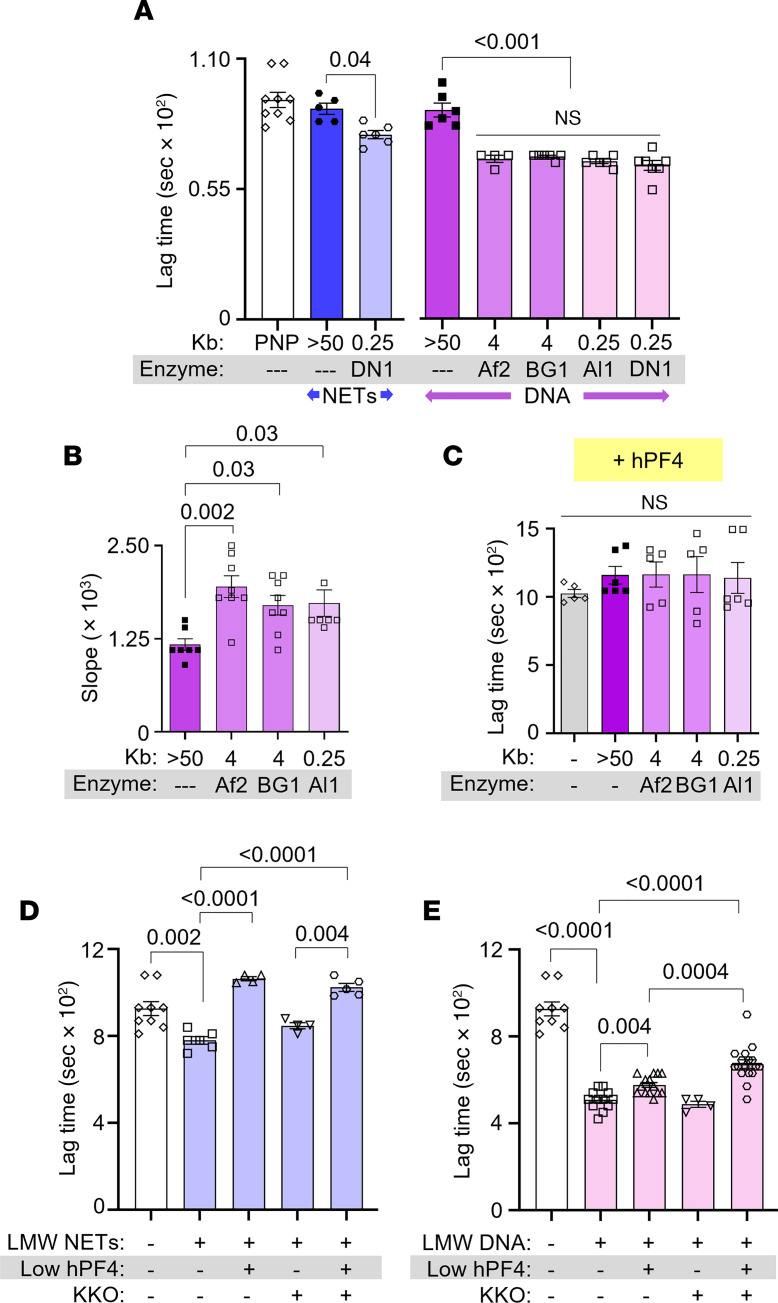
hPF4 and KKO inhibit fibrin generation initiated by LMW NETs and DNA. Fibrin generation in pooled normal plasma (PNP) with or without added HMW or LMW NETs (blue) or DNA (purple). DN1, DNase I; Af2, AflII; BG1, BsrGI-HF; Al1, AluI. Data are mean ± SEM of at least 3 independent experiments. (**A**) Lag time determined from kinetic curves of fibrin generation with NETs (blue) and DNA (purple). (**B**) Slope (rate) of fibrin generation was determined from the same kinetic curves as in **A**. (**C**) Lag time of DNA-induced fibrin generation was determined from kinetic curves similarly to **A**, in the presence of hPF4 (20 μg/mL). (**D** and **E**) Lag times are shown for fibrin generation studies of DNase I–digested LMW NETs (**D**) and DNA (**E**) in the absence or presence of low hPF4 (1 μg/mL) and/or KKO (10 μg/mL). Data are mean ± SEM of at least 3 independent experiments. Comparative statistical analysis was performed by Kruskal-Wallis 1-way ANOVA.

We treated HMW NETs and DNA of greater than 50 kilobase pairs (kb) (Supplemental Figure 1A; supplemental material available online with this article; https://doi.org/10.1172/jci.insight.171054DS1) with DNase I to generate fragments of LMW NETs and DNA measuring 0.1–0.5 kb (Supplemental Figure 1B). To determine whether fragment length influences DNA-mediated fibrin generation, HMW DNA (>50 kb) was also digested with 6-cutter restriction enzymes, AflII and BsrGI-HF, and the 4-cutter, AluI, to generate DNA fragment lengths of approximately 4 kb and approximately 250 bp, respectively (Supplemental Figure 1C). We then assessed the thrombogenicity of these NETs and DNA in plasma using a fibrin generation assay, measuring time to initiation of fibrin generation (lag time), the rate of fibrin generation (slope), and peak fibrin formation. HMW NETs and DNA did not accelerate fibrin generation in plasma (Figure 1A). However, the addition of LMW NETs shortened lag time from approximately 900 to approximately 720 seconds (Figure 1A), while DNA fragments of various lengths cleaved by restriction enzymes all significantly shortened lag time to approximately 650 seconds (Figure 1A) and increased the rate (slope) of fibrin generation by approximately 40% (Figure 1B). As expected, there was no difference in peak fibrin formation between groups (data not shown).

### hPF4 and KKO inhibit fibrin generation initiated by NETs and DNA.

The addition of 20 μg/mL hPF4, a concentration achieved locally at sites of thrombus formation (41), prolonged LMW-DNA-induced fibrin generation lag time, regardless of DNA fragment length (Figure 1C). hPF4 alone had no effect on lag time in plasma in the absence of DNA. At a lower concentration (1 μg/mL), hPF4 still fully reversed the prothrombotic effects of LMW NETs (Figure 1D) and was partially effective in the case of LMW DNA (Figure 1E). The difference between NETs and DNA could be due to the presence of positively charged histones bound to the polyphosphate backbone of NETs.

Although we previously observed that binding of KKO to hPF4-NET complexes increased the resistance of NETs to nuclease digestion (32), inclusion of KKO (10 μg/mL) did not enhance the ability of hPF4 at concentrations between 2.5 and 20 μg/mL to delay LMW-DNA-induced fibrin generation (Supplemental Figure 2A); however, when the hPF4 concentration was decreased to 1 μg/mL, the addition of KKO (10 μg/mL) to LMW NETs and DNAs significantly prolonged fibrin lag time (Figure 1E and Supplemental Figure 2B). These results indicate that hPF4 interacts with NETs and DNA to reduce their fibrin generation potential in human plasma, an effect that is enhanced by KKO binding to the hPF4-containing complexes.

### hPF4 inhibits thrombin generation, but not fibrinolysis, by NETs and DNA.

**Figure 2:**
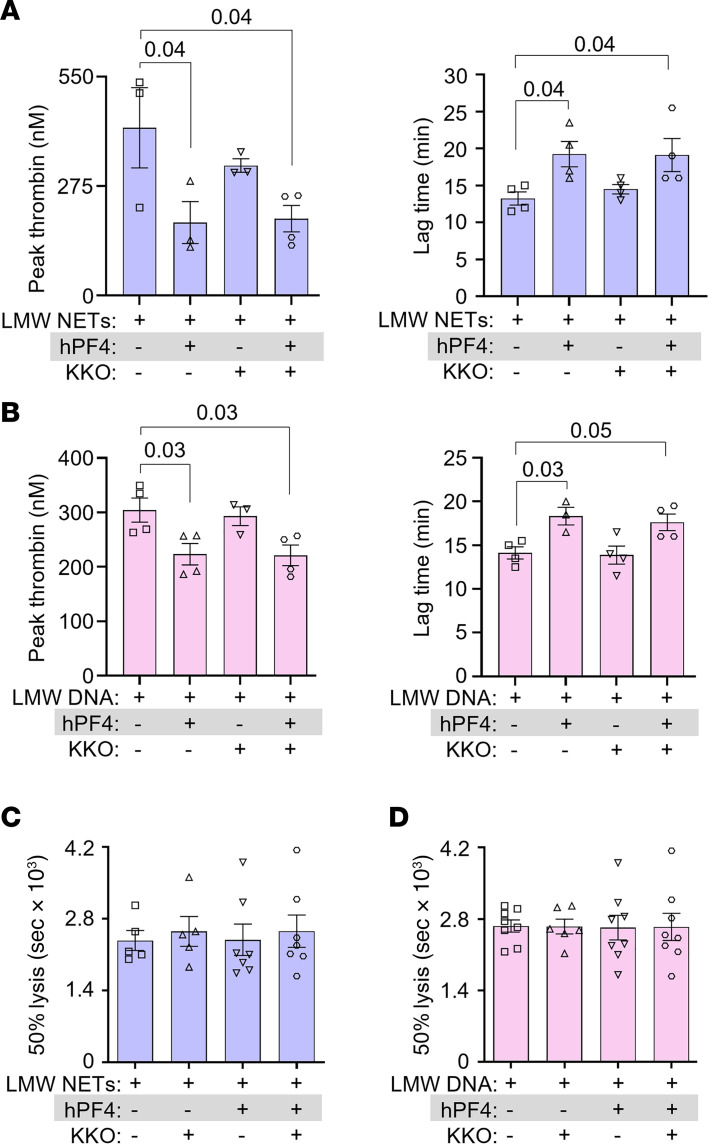
hPF4 and KKO inhibit thrombin generation but not fibrinolysis. (**A** and **B**) Thrombin generation studies of DNase I–digested LMW NETs (**A**) and DNA (**B**) in the absence or presence of hPF4 (20 μg/mL) and/or KKO (10 μg/mL). (**C** and **D**) Time to 50% clot lysis of LMW NET–induced (**C**) and LMW DNA–induced (**D**) coagulation, as determined based on kinetic curves. Data are mean ± SEM of at least 3 independent experiments. Comparative statistical analysis was performed by Kruskal-Wallis 1-way ANOVA.

We next measured thrombin generation to address the mechanism by which hPF4 and KKO inhibited the procoagulant effects of LMW NETs and DNA. hPF4 significantly reduced NET-induced peak thrombin generation and delayed thrombin initiation time (Figure 2, A and B, respectively). hPF4 also significantly reduced DNA-induced thrombin generation and delayed thrombin lag time (Figure 2, A and B). The addition of KKO did not alter hPF4-mediated inhibition of NET- or DNA-induced thrombin generation (Figure 2, A and B, and Supplemental Figure 3A).

Enhanced coagulability and defects in fibrinolysis lead to an increased risk of large vessel thrombosis in septic patients. There have been conflicting reports concerning the effect of NETs and cfDNA on thrombus stability. Some studies showed NET potentiation of fibrinolysis by stimulating fibrin-independent plasminogen activation. Others observed that NET components, such as neutrophil elastase and cfDNA, promoted resistance to thrombolysis by degrading plasminogen and forming lysis-resistant complexes with fibrin, respectively (42, 43). Therefore, we assessed whether hPF4-NET or hPF4-DNA complexes affect the rate of internal fibrinolysis in NET- and DNA-induced plasma clots by mixing tissue-type plasminogen activator (tPA) with LMW NETs or DNA with or without added hPF4 prior to recalcification. While hPF4 (1 and 20 μg/mL) and KKO (10 μg/mL) delayed LMW-NET- and LMW-DNA-induced fibrin generation significantly as described above, we observed no difference in clot lysis time (Figure 2, C and D) at the concentrations used. These findings indicate that hPF4, which prevents DNases from digesting NETs (32), does not directly interfere with fibrinolysis.

### hPF4 modulates fibrin formation through the intrinsic coagulation pathway.

**Figure 3:**
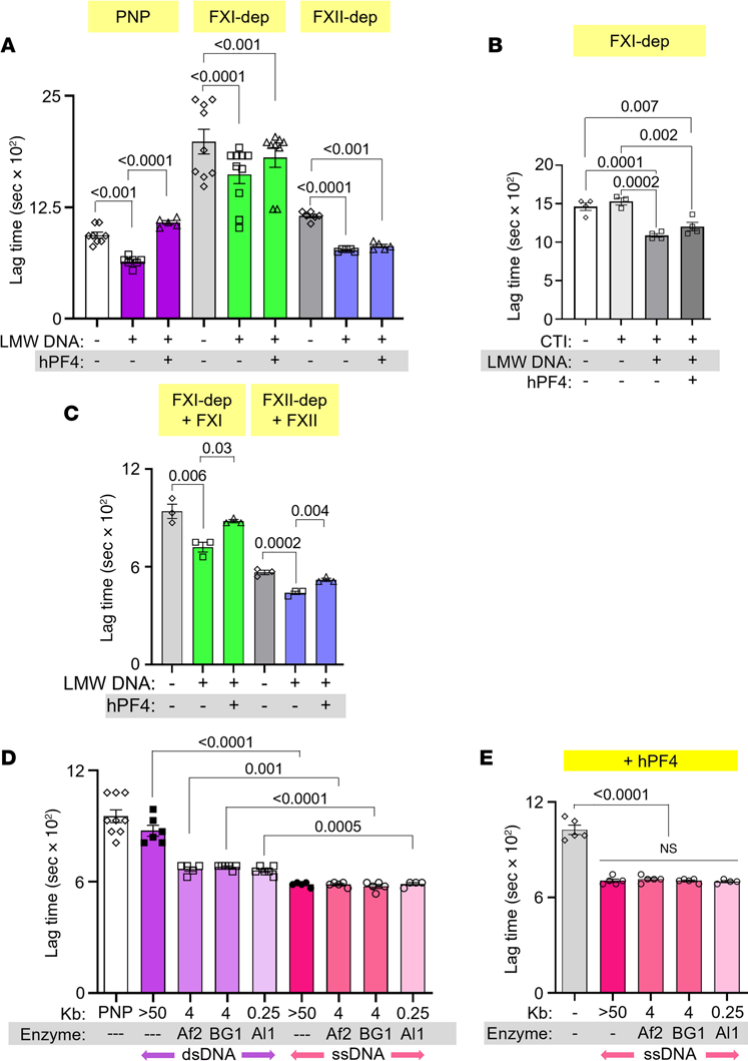
Effect of hPF4 on fibrin generation in FXI- and FXII-depleted plasma and on thrombogenicity of ssDNA. (**A**) Lag time, determined from kinetic curves, of fibrin generation in PNP or FXI- or FXII-depleted (dep) plasma, induced by DNase I–digested LMW DNA in the absence or presence of hPF4. (**B**) Fibrin generation lag time in FXI-depleted plasma spiked with CTI, induced by LMW DNA in the absence or presence of hPF4. (**C**) Lag time of fibrin generation induced by LMW DNA in depleted plasma supplemented with missing coagulation factors. (**D**) Lag time of ssDNA-induced (pink) fibrin generation compared to dsDNA (purple). (**E**) Lag time of ssDNA-induced fibrin generation with added hPF4 (20 μg/mL). Data are mean ± SEM of at least 3 independent experiments. Comparative statistical analysis was performed by Kruskal-Wallis 1-way ANOVA.

We next sought to define the mechanism by which hPF4 modulates the prothrombotic effects of LMW DNA. Polyanions, such as polyphosphates and DNA, trigger thrombin generation in plasma by activating the intrinsic pathway of coagulation, converting prekallikrein to kallikrein, which cleaves factor IX (FIX), leading to fibrin generation (44–47). In fibrin generation studies using FXI- and FXII-depleted plasma, the presence of DNA still induced shortening of lag time (Figure 3A and Supplemental Figure 3B), perhaps because DNA triggers FXI autoactivation independently of FXII (45), and pathological FXII activation is thrombogenic even in an FXI-independent manner (46). We then spiked corn trypsin inhibitor (CTI) into FXI-depleted plasma to inhibit FXII activity, and observed that LMW DNA also shortened lag time (Figure 3B). This is consistent with previous reports showing that DNA activates prekallikrein into kallikrein, which then cleaves FIX and leads to fibrin generation (48, 49). The addition of hPF4, at concentrations as high as 20 μg/mL, had no effect on LMW-DNA-induced fibrin generation in the absence of FXI, FXII alone, or both FXI and FXII (Figure 3, A and B, and Supplemental Figure 3B). Upon supplementation of FXI-depleted plasma with FXI, hPF4 significantly delayed DNA-induced fibrin generation lag time (Figure 3B and Supplemental Figure 4A). hPF4 also delayed fibrin generation in FXII-depleted plasma supplemented with FXII (Figure 3C and Supplemental Figure 4B). These results suggest that hPF4 blocks the procoagulant activity of LMW DNA in plasma, at least in part, by interfering with DNA-mediated activation of the intrinsic pathway of coagulation.

### hPF4 attenuates the thrombogenicity of ssDNA.

We hypothesized that LMW NETs and DNA fragments are more thrombogenic than HMW NETs and DNA because they exhibit greater levels of ssDNA at their termini and form more hairpin structures, both potent activators of coagulation (50). To investigate this hypothesis, we denatured DNA fragments of different size ranges at 100°C for 10 minutes and then rapidly froze the samples to generate ssDNA fragments of various lengths (51). ssDNA fragments of all lengths dramatically accelerated fibrin generation, an interval significantly shorter than lag times seen with either HMW or LMW double-stranded DNA (dsDNA) (Figure 3D). hPF4 delayed fibrin lag time by ssDNA, but not to the same extent as seen with dsDNA (Figure 3D versus Figure 3E), perhaps because ssDNA exhibits fewer sites for hPF4 binding.

### hPF4 protects endothelium from activation of prothrombotic pathways by DNA.

**Figure 4:**
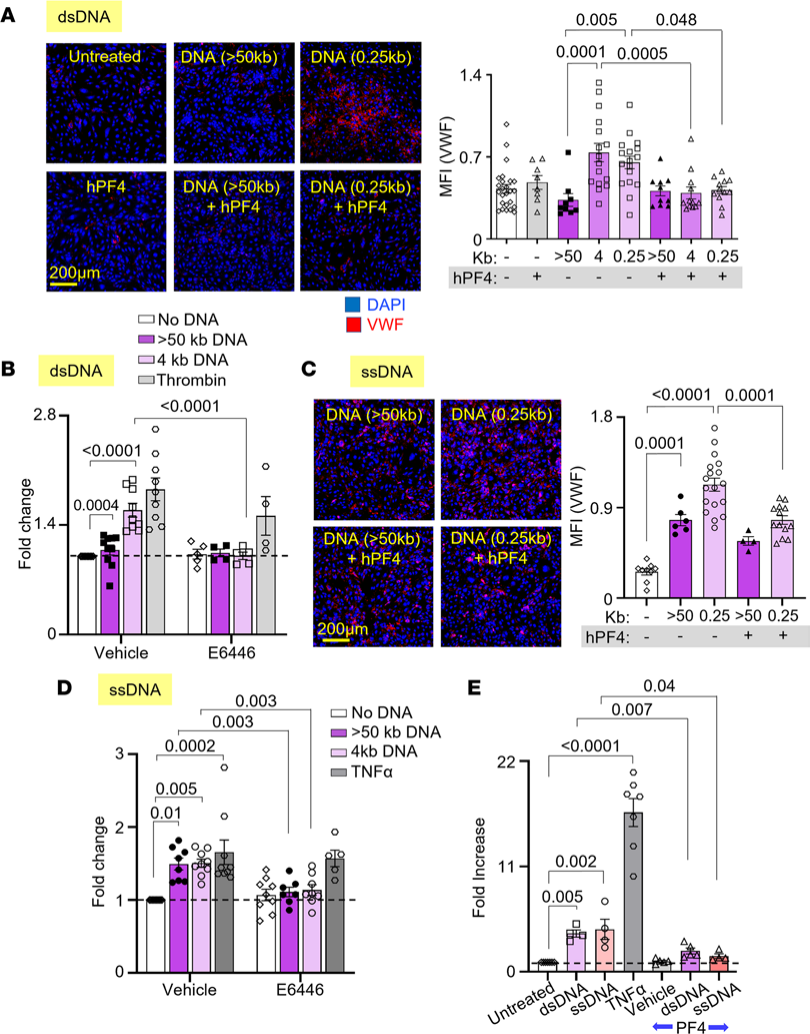
PF4 protects against LMW dsDNA– and ssDNA–induced procoagulant responses by endothelium. Mean fluorescence intensity (MFI) of released VWF from HUVECs exposed to fragments of dsDNA (**A** and **B**) or ssDNA (**C** and **D**), with or without hPF4 or TLR9 inhibitor (E6446). Data were normalized to MFI of untreated cells without exposure to dsDNA or inhibitor (as indicated by dotted lines). Exposure to antithrombin (**B**) or TNF-α (**D**) serves as positive controls. (**E**) TF expression by HUVECs (shown as fold increase from untreated cells without PF4 exposure) induced by dsDNA or ssDNA in the absence or presence of hPF4. TNF-α exposure was used as a positive control. Scale bars: 200 μm. Data are mean ± SEM of at least 3 independent experiments. Comparative statistical analysis was performed by Kruskal-Wallis 1-way ANOVA.

Our group previously found that hPF4 decreases NET-mediated endothelial toxicity (32). Building on this observation, we now assessed whether hPF4 modulates the ability of LMW DNA to promote a procoagulant phenotype by endothelial cells. Human umbilical vein endothelial cells (HUVECs) were incubated with DNA fragments of different size ranges with and without hPF4, prior to staining for VWF release as an indicator of HUVEC activation. HMW DNA did not induce VWF secretion by HUVECs, while LMW dsDNA fragments (0.25–4 kb) significantly increased surface VWF to levels similar to those seen upon activation by thrombin (52) (Figure 4, A and B, and Supplemental Figure 5A). hPF4 prevented dsDNA-induced VWF secretion regardless of DNA length (Figure 4A and Supplemental Figure 5A). Prior studies showed that internalized, unmethylated CpG motifs in DNA activate endothelial cells via TLR9 (53). We now treated cultured HUVECs with TLR9 inhibitors, E6446 and hydroxychloroquine (HCQ), and found that each inhibitor abolished dsDNA-induced VWF secretion (Figure 4B and Supplemental Figure 5C).

Unlike HMW dsDNA, HMW ssDNA stimulated robust VWF release comparable to levels of release seen with LMW dsDNA. ssDNA fragments induced VWF release at nearly twice the level seen with similarly sized dsDNA and comparable to that seen using exposure to TNF-α (54) (Figure 4, C and D, and Supplemental Figure 5B). hPF4 significantly reduced endothelial release of VWF by ssDNA, although not as effectively as after exposure to dsDNA (Figure 4C). Inhibition of TLR9 with E6446, and to a lesser extent HCQ, reduced ssDNA-induced VWF secretion (Figure 4D and Supplemental Figure 5D).

NETs induce endothelial TF expression (15), but the NET component(s) that are responsible for this effect have not been defined. To determine whether NET cfDNA upregulates endothelial TF expression, HUVECs were incubated with LMW dsDNA and ssDNA fragments overnight prior to exposure to FX and activated FVII (FVIIa). Rate of cleavage of a chromogenic substrate by activated FX (FXa) was measured as an indicator of endothelial TF expression and activity. Both LMW dsDNA and ssDNA fragments enhanced TF expression approximately 4.5-fold compared with vehicle alone, while the inclusion of hPF4 during overnight incubation significantly reduced endothelial TF expression induced by both (Figure 4E).

### Infused HMW DNA increases plasma TAT levels in Cxcl4^–/–^ mice.

**Figure 5:**
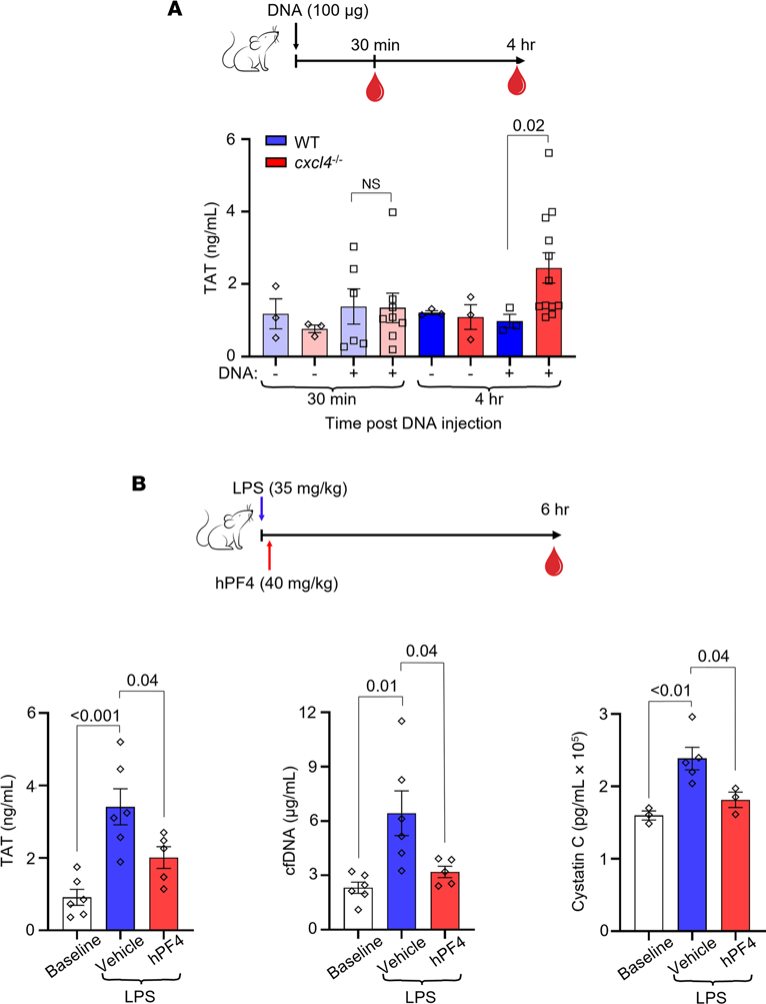
hPF4 attenuates cfDNA-induced coagulability and organ damage in vivo. (**A**) Top: Schematic of study in which WT mice or *Cxcl4*^–/–^ littermates were given normal saline vehicle or normal saline containing digested DNA prior to blood collection at 30 minutes or 4 hours. Bar graph: TAT levels using a commercial ELISA kit in platelet-poor plasma. Data are mean ± SEM of at least 3 independent experiments. (**B**) Top: Schematic of study in which WT mice received normal saline vehicle or LPS, and a subset of animals was given hPF4 by tail vein injection immediately following LPS injection. TAT, cfDNA, and cystatin C levels were measured in blood drawn at baseline and 6 hours after LPS with and without hPF4 infusion. Bar graphs: TAT levels (left), cfDNA levels (middle), and cystatin C levels (right) 6 hours after LPS challenge. Data are mean ± SEM of at least 5 independent experiments. Comparative statistical analysis was performed by Kruskal-Wallis 1-way ANOVA.

To assess the thrombogenicity of cfDNA in vivo and to define the role of PF4 in modulating DNA-induced thrombosis, wild-type (WT) or *Cxcl4^–/–^* littermate mice were given an intravenous (i.v.) bolus of HMW DNA, and plasma levels of TAT complexes were measured. Although there was no difference in TAT levels between WT and *Cxcl4^–/–^* mice 30 minutes after DNA infusion, *Cxcl4^–/–^* mice had significantly elevated TAT levels compared with WT mice 4 hours following DNA injection (Figure 5A), consistent with the in vitro thrombotic effects of DNA on endothelial cells seen in Figure 4.

### hPF4 inhibits thrombin generation by cfDNA in endotoxemic mice.

To determine whether hPF4 modulates the thrombogenicity of cfDNA released during sepsis, WT mice were given an intraperitoneal (i.p.) injection of LPS to induce endotoxemia, an intervention previously shown by us and others to increase plasma cfDNA levels (22, 32). A subset of these mice was given saline vehicle or hPF4 by tail vein injection immediately following LPS injection. Six hours after LPS injection, saline-treated mice exhibited a significant elevation in TAT, cfDNA, and cystatin C levels (Figure 5B). In contrast, mice administered hPF4 had significantly lower levels of TAT, cfDNA, and cystatin C (Figure 5B), consistent with protective effects of hPF4 against thrombosis, endothelial cell injury, and end-organ dysfunction.

## Discussion

**Figure 6:**
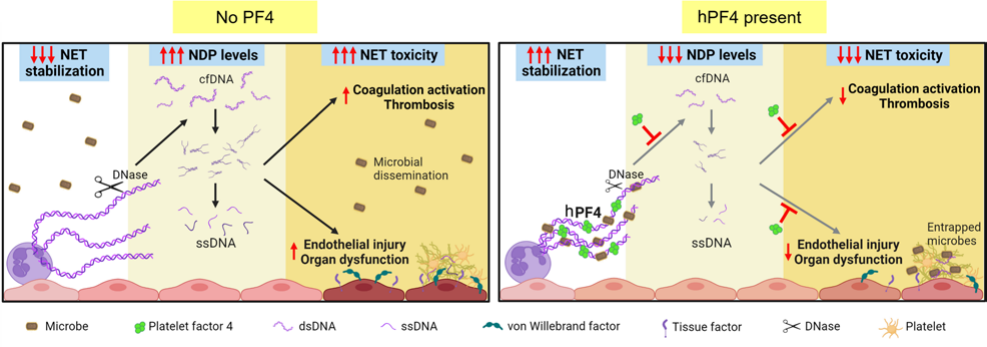
Proposed protective mechanisms of NET stabilization. Left: NETs are subjected to digestion by DNase I, reducing microbial capture and liberating toxic NDPs, including cfDNA. These cfDNA fragments expose ssDNA at termini that trigger coagulation activation and induce endothelial injury, leading to thrombosis and end-organ dysfunction in sepsis. Right: NET stabilization by hPF4 enhances DNase I resistance, which enhances NET microbial capture, reduces circulating cfDNA levels, and attenuates cfDNA-induced thrombogenicity and toxicity to endothelial cells.

It may seem counterintuitive to propose NET stabilization as a therapeutic strategy, as elevated NET markers have been associated with increased disease severity in a diverse range of thromboinflammatory disorders, including sepsis, COVID-19, and sickle cell disease (16, 17, 55–75). However, studies from multiple groups show that the formation of NETs is an evolutionarily conserved function, initially noted in protozoa and retained as part of the innate immune response of plants and animals to capture invading pathogens and immobilize them near concentrated antimicrobial compounds (76). Our data and those of others (9, 10, 31, 77, 78) support the notion that intact NETs are not pathogenic when released in a regulated manner. Intact NETs may limit collateral host tissue damage by preventing the systemic release of toxic NDPs, including cfDNA, which activates the contact coagulation pathway and acts as a DAMP (Figure 6).

Developing a NET-based therapeutic approach in sepsis remains an unmet clinical need. Others have previously proposed treating sepsis by blocking NETosis (21, 79–90) or accelerating NET degradation (91–102); however, neither strategy enhances the beneficial effects of NETs. The latter liberates entrapped pathogens and increases plasma cfDNA levels. Our group previously showed that hPF4 binds to NETs, causing them to become physically compact and resistant to nucleases, limiting the release of NDPs (32). Moreover, the positively charged hPF4 tetramer, found in high molar concentrations at sites of platelet degranulation, cross-binds negatively charged microbes to the negatively charged DNA scaffold of NETs. Without hPF4, microbial entrapment is markedly impaired (32). That study supported a model in which hPF4 exerts positive effects on NETs, preventing excessive NET degradation and enhancing microbial entrapment. In this report, we show that hPF4 dampens the thrombotic and endothelial cell–activating effects of cfDNA fragments derived from HMW DNA, demonstrating that hPF4 may also exert protective effects in sepsis by aggregating circulating nucleic acids that originate not only from NETs, but from necrosis, apoptosis, and increased red cell turnover.

We posited that, regardless of its source, smaller fragments of DNA have a higher thrombotic potential because they have a greater number of termini that can “breathe,” exposing thromboinflammatory ssDNA (103). To explore this idea, we showed that HMW DNA, like HMW NETs, has limited thrombogenicity and ability to stimulate the endothelium. This finding differs from a prior report of DNA’s effect on thrombogenicity, likely due to their use of only LMW DNA (40). We also generated ssDNA by melting HMW DNA and observed that irrespective of fragment length, ssDNA was more prothrombotic and injurious to the endothelium than dsDNA. It is likely that shorter dsDNA fragments, cleaved from digested NETs, expose more ssDNA that can form noncanonical secondary structures such as hairpin loops that can avidly bind kininogen, serving as potent activators of the intrinsic coagulation pathway (50). Moreover, ssDNA internalized by endothelial cells binds to TLR9 to induce a prothrombotic phenotype (104). It is increasingly recognized that ssDNA is present in human plasma (105, 106) and is revealed on the jagged ends of cfDNA following cleavage by DNase I and DNase IL3 (107), supporting the physiologic relevance of our findings.

The hPF4 tetramer may be able to combat the prothrombotic effects of cfDNA because of its positively charged circumferential zone that can bind to 2 polyanions, 1 on each side (108). This bivalent property enables a single tetramer of hPF4 to simultaneously bind the polyphosphate-ribose backbone of 2 separate DNA chains, leading to NET compaction and cfDNA aggregation. Our in vitro studies show that hPF4, at concentrations that are well within the range seen in the setting of thromboinflammation (41), can reduce the prothrombotic and endothelial cell–activating properties of LMW dsDNA and ssDNA. Our in vivo experiments in mice infused with genomic DNA or treated with LPS similarly demonstrate that the expression of hPF4 protects animals from plasma-cfDNA-enhanced thrombosis, endothelial activation, and organ injury, consistent with our model (Figure 6) and our prior studies that showed treatment with hPF4 improved outcomes in both murine LPS endotoxemia and cecal ligation and puncture–induced polymicrobial sepsis (32, 35).

Of note, further stabilization of hPF4-NET complexes with KKO does not interfere with hPF4’s ability to reduce procoagulant activity and endothelial cell activation, nor does it impair thrombolysis. Indeed, hPF4 and KKO likely work in concert to reduce the pathogenic effects of NETs and cfDNA by further protecting against DNase fragmentation (32). While KKO does not exert an additive antithrombotic effect when hPF4 is present at high concentrations (≥10 μg/mL), levels that only develop locally at sites of intense platelet activation, it augments the effect of lower concentrations of hPF4 (1 μg/mL) that are more likely to be seen in the systemic circulation. Because platelet counts and hPF4 content per platelet vary in the general population (109, 110) and critically ill individuals often develop thrombocytopenia (111, 112), we propose that septic patients may benefit from either hPF4 and/or Fc-modified KKO infusion to optimize NET stabilization.

In summary, NETs have an important positive role in host defense, impeding the spread of pathogens and limiting the systemic release of toxic antimicrobial compounds. However, when degraded, NETs release LMW NETs and DNA that can expose ssDNA, initiating the contact pathway of coagulation and activating the endothelium, in part, via TLR9. We previously showed that hPF4 blocks NET fragmentation and enhances its entrapment of microbes. We now show that hPF4 limits the procoagulant and endothelium-stimulating effects of LMW NETs and DNAs. KKO, which further decreases NET susceptibility to nucleases, does not interfere with hPF4’s ability to prevent pathologic thrombosis and endothelial activation. Indeed, KKO enhances these effects at lower hPF4 concentrations. These studies support further investigation of NET stabilization strategies to enhance the protective effect of intact, HMW NETs, while limiting the toxicities of plasma LMW DNA.

## Methods

### Supplemental materials and methods.

See the Supplemental Materials and Methods for further details on reagents, preparation of recombinant hPF4, isolation of human neutrophils, i.v. administration of DNA into mice, as well as mouse cystatin C ELISA.

### Preparation of HMW and LMW NETs.

Purified human neutrophils (2 × 10^7^ to 3 × 10^7^) were plated on a 10-mm petri dish for 30 minutes at 37°C. The neutrophils were then treated with phorbol-2-myristate-13-acetate (100 nM) for 4 hours at 37°C. Cells were washed with phosphate-buffered saline (PBS). A low dose of DNase I (4 U/mL) was added for 20 minutes at 37°C to cleave intact NETs from cell bodies with minimal DNA degradation, yielding HMW NETs greater than 50 kb long (Supplemental Figure 1A). EDTA (5 mM final; Thermo Fisher Scientific) was added to inactivate the DNase I. The supernatant containing HMW NETs was centrifuged at 3000*g* for 10 minutes at 4°C to remove cell debris, and then collected and stored at –20°C. Nucleic acid concentration was measured using a Nanodrop spectrophotometer (Thermo Fisher Scientific) as per the manufacturer’s instructions. To generate LMW NETs, HMW NETs were incubated with DNase I (100 U/mL) for 2 hours at 37°C to yield fragments 0.1–0.5 kb long (Supplemental Figure 1B). EDTA (5 mM final) was added after completion of digestion to inactivate DNase I.

### Extraction of HMW DNA from human neutrophils.

Purified human neutrophils from 50 mL of whole blood were resuspended in Hank’s balanced salt solution (HBSS) prior to cell lysis with 10 mM Tris HCl (pH 8), 10 mM EDTA, 10 mM NaCl, and 0.5% SDS, and incubated with proteinase K (10 mg/mL; Roche) at 37°C overnight, as described previously (113, 114). An equal volume of ultrapure phenol/chloroform/isoamyl alcohol (25:24:1, Thermo Fisher Scientific) was added with vigorous swirling to mix for 15 minutes at room temperature (RT), and then centrifuged at 3000*g* for 10 minutes at 4°C. The aqueous supernatant was gently transferred to a new tube containing 2.5× volume of 100% ethanol (Pharmco), and the layers were swirled slowly to precipitate DNA. Strands of genomic DNA were removed from solution using a clean glass rod. The DNA was washed with 70% ethanol and centrifuged at 3000*g* for 10 minutes at RT. The DNA pellet was air dried and resuspended in Tris-EDTA buffer (pH 8) overnight at 4°C, and then stored at –20°C. Nucleic acid concentration was measured using the Nanodrop spectrophotometer, with an A260/280 ratio of approximately 1.8 as an indication of pure DNA.

### Preparation of LMW dsDNA and ssDNA.

Isolated HMW DNAs were digested into shorter fragments by incubating with restriction enzymes AflII, BsrGI-HF, or AluI (10 U/mg DNA in CutSmart buffer, all from New England BioLabs) overnight at 37°C, or DNase I (100 U/mL in PBS, Gibco) for 2 hours at 37°C. ssDNA was generated by heating HMW or LMW dsDNA at 100°C for 10 minutes, followed by snap freezing on dry ice to prevent re-annealing, as previously described (51).

### Characterization of HMW and LMW NETs and DNA.

NET and DNA fragments were size fractionated in ethidium bromide–stained (0.5 μg/mL) agarose gels (0.4%–0.9% agarose, GeneMate) in Tris–acetic acid EDTA buffer (40 mM Tris, 1 mM EDTA, 20 mM acetic acid) along with high-range (>50 kb), 1 kb, and 100 bp DNA markers. Bands were visualized under UV light as previously described (113).

### Thrombin generation assay.

For the thrombin generation assay (TGA), HMW and LMW NETs (20 μg/mL) and DNA (20 μg/mL) were incubated with or without hPF4 (20 μg/mL) and KKO (20 μg/mL) in buffer containing a final concentration of 4 μM phosphatidylcholine/phosphatidylserine (75:25; Diapharma) for 10 minutes at RT in 96-well black MaxiSorp plates (475515, NUNC). Pooled normal human plasma (33% assay volume; George King Bio-Medical) was added, followed by Technothrombin TGA substrate Z-GLY-GLY-ARG-AMC • HCl (417 μM substrate, 6.25 mM CaCl_2_ final concentration, Bachem Holding) in TGA dilution buffer (20 mM HEPES, 150 mM NaCl, 0.1% PEG-8000; pH 7.4). Fluorescence at 360 nm/460 nm excitation/emission (ex/em) was measured immediately and at 1-minute intervals for 90 minutes at 37°C. Thrombin generation was calculated against a thrombin calibration curve (calibration kit, Diapharma).

### Fibrin generation assay.

HMW and LMW NETs (20 μg/mL) and DNA (20 μg/mL) were incubated with or without hPF4 (20 μg/mL) and KKO (20 μg/mL) for 10 minutes at 37°C in HBS buffer (25 mM HEPES, 150 mM NaCl; pH 7.4) in clear, medium-binding 96-well plates (9017, Corning). Pooled normal human plasma, FXI-depleted, or FXII-depleted plasma (33% assay volume; George King Bio-Medical) was added, followed by 8.3 mM CaCl_2_ in HBS buffer. In some experiments, FXI-depleted plasma was spiked with CTI (50 μg/mL) to block FXII activity. Absorbance at 405 nm was measured for 1 hour at 30-second intervals at 37°C. Lag time (after recalcification until first burst of fibrin generation) and slope (steepest rate of fibrin generation/min) were determined from kinetic curves. In select experiments, depleted plasma was supplemented with purified FXI (100 pM) or FXII (1.2 nM) (Haematologic Technologies) prior to recalcification and fibrin generation.

### Fibrinolysis assay.

NETs (2 μg/mL) and dsDNA (2 μg/mL) were incubated with or without hPF4 (20 μg/mL) and KKO (10 μg/mL) prior to adding tPA (40 ng/mL; Diapharma) in 96-well plates (Corning). Plasma (33% assay volume) was immediately added and recalcified (15 mM CaCl_2_ final concentration) as described above. Absorbance at 405 nm (A405) was measured for 2 hours at 1-minute intervals at 37°C. The 50% lysis time was defined as the time between half-maximal A405 on the ascending curve and half-maximal A405 on the descending curve.

### HUVEC activation studies.

HUVECs (Lifeline Cell Technology, FC-0044) were grown to confluence on 0.1% gelatin–coated (ATCC) glass-bottom 8-well Ibidi chambers with Vasculife VEGF endothelial medium complete kit (Lifeline). HUVECs were incubated with vehicle (serum-free media, SFM; Gibco) or dsDNA (20 μg/mL) or ssDNA (20 μg/mL) with or without hPF4 (20 μg/mL) added for 20 minutes at 37°C. In select experiments, HUVECs were pretreated with TLR9 inhibitor E6446 (10 μM; Selleckchem) or HCQ (50 μM; Selleckchem) for 30 minutes prior to DNA exposure with or without hPF4, as described above. Human antithrombin (10 nM; Haematologic Technologies) and TNF-α (20 ng/mL; R&D Systems) were used as positive controls.

To measure VWF release, HUVECs were washed with PBS and fixed in 4% paraformaldehyde (Santa Cruz Biotechnology) for 15 minutes at RT before blocking with 3% bovine serum albumin and 2% fetal bovine serum (HyClone) in PBS for 1 hour at RT. Primary anti–human VWF antibody (1:1000 dilution in PBS; Agilent Dako, A0082) was incubated overnight at 4°C. Slides were washed with PBS and blocked for 1 hour at RT. Secondary Alexa Fluor 594 anti–rabbit IgG (4 μg/mL; Invitrogen, A32740) and Hoechst 33342 (10 μg/mL; Invitrogen, H3570) were incubated in PBS for 2 hours at RT in the dark. HUVECs were imaged using Zeiss 10× and 20× objectives on a Zeiss LSM 710 confocal microscope. Mean fluorescence intensity (MFI) was quantified using Fiji (NIH) open-source image processing software (115).

To measure TF surface expression and activity, we utilized a previously established technique to measure FXa generation, as TF-FVII complexes bind and activate FX (116, 117). HUVECs were grown to confluence in tissue culture–treated 96-well plates. Cells were washed with SFM and incubated with vehicle, digested dsDNA or ssDNA (each 20 μg/mL), and TNF-α (1 ng/mL) for 16 hours in SFM in the absence or presence of hPF4 (20 μg/mL). Cells were then washed with assay buffer (20 mM Tris, 100 mM NaCl, 10 mM CaCl_2_, pH 7.4). FX (160 nM) and FVIIa (0.5 nM; Enzyme Research Labs) were added to the assay buffer and incubated for 1 hour at 37°C. SPECTROZYME Xa (0.5 mM, BioMedica Diagnostics) was added and A405 was immediately recorded for 1 hour at 1-minute intervals to determine the maximum rate of reaction, *V*_max_ (mOD/minute).

### LPS endotoxemia model.

WT C57BL/6J mice (Jackson Laboratory, strain 000664) received an i.p. injection of LPS (35 mg/kg). A subset of animals was given 40 mg/kg of hPF4 by tail vein injection immediately thereafter. Six hours following LPS injection, animals were euthanized, blood was collected from the inferior vena cava into 3.2% sodium citrate (1:10 v/v), and platelet-poor plasma was isolated by centrifugation at 2000*g* for 20 minutes at RT.

To assess thrombin generation in murine septic plasma, TAT complexes were measured by ELISA kit (AssayPro, EMT1020-1) as follows: citrated plasma was diluted 125-fold and incubated for 2 hours at RT in 96-well plates precoated with polyclonal antibody against mouse thrombin. The wells were washed and incubated with biotinylated mouse TAT complex antibody for 1 hour at RT. The microplate was then incubated with streptavidin-peroxidase conjugate for 30 minutes at RT, and chromogenic substrate was then added for 5 minutes at RT. Reactions were stopped and absorbance was immediately measured at 450 nm.

cfDNA levels were measured in the plasma from LPS-treated mice, diluted 1:10 with HBSS (no Ca^+2^/Mg^+2^; Gibco) in a black MaxiSorp 96-well plate. An equal volume of SYTOX Green (5 μM final in HBSS without Ca^+2^/Mg^+2^; Invitrogen, S7020) was added to each sample and incubated in the dark for 10 minutes. Fluorescence at 485/527 ex/em was measured at RT, and plasma cfDNA concentrations were determined based on fluorescence of calf thymus DNA curve of 0 to 10 μg/mL. To assess kidney injury, cystatin C levels were measured by ELISA kit (R&D Systems, DY1238) according to the manufacturer’s instructions.

### Statistics.

Data are presented as mean ± standard error of the mean (SEM). The Shapiro-Wilk normality test was used to determine whether group data were distributed normally. Levene’s test was used to determine equality of variances. One-way ANOVA with Tukey’s post hoc test was used to compare between treatment groups. Mann-Whitney test or Kruskal-Wallis with Dunn’s post hoc test was used to compare between groups when the data did not qualify for parametric statistics. A *P* value of 0.05 or less was considered significant. All statistical analyses were conducted using GraphPad Prism 9.

### Study ethics approval.

Human blood for studies was collected after informed consent from healthy, aspirin-free volunteers using a 19-gauge butterfly needle in 3.8% sodium citrate (10:1 v/v) under a protocol approved by the Children’s Hospital of Philadelphia Institutional Review Board and were consistent with the Principles of Helsinki. Animal procedures were approved by the Institutional Animal Care and Use Committee (IACUC) in accordance with the NIH *Guide for the Care and Use of Laboratory Animals* (National Academies Press, 2011) and the Animal Welfare Act.

### Data availability.

Original data are available in the Supporting Data Values XLS file.

## Author contributions

ATPN contributed to experimental design, performed experiments, analyzed and evaluated data, and wrote the manuscript. A Skidmore, JO, IY, NL, VB, and A Sarkar performed experiments and edited the manuscript. GZ, LR, MAK, KE, NSM, and AR contributed to mouse breeding, experimental design, and data interpretation. DBC contributed to discussions of the proposed research and to editing the manuscript. MP and KG conceived, designed, and supervised the project and contributed to writing of the manuscript.

## Supplementary Material

Supplemental data

Supporting data values

## Version 1 11/22/2023

Electronic publication
