# Supplementary material for: Platelet factor 4 limits neutrophil extracellular trap– and cell-free DNA–induced thrombogenicity and endothelial injury
Source: JCI Insight. 2023 Nov 22;8(22):e171054. doi: 10.1172/jci.insight.171054 (PMC10721321; doi:10.1172/jci.insight.171054)
Supplement: Supplemental data [file jciinsight-8-171054-s249.pdf]

SUPPLEMENTAL MATERIALS AND METHODS

Reagents

All reagents were purchased from Sigma-Aldrich except as noted. Recombinant hPF4 was expressed in S2 cells and purified using affinity chromatography and protein liquid chromatography as previously described (1). The end-product was found to be endotoxin-free using ToxinSensor™ chromogenic LAL endotoxin assay kit (Genscript L00350), and was tested for size distribution by SDS-polyacrylamide gel electrophoresis. KKO is a mouse $IgG_{2b\kappa}$ anti-hPF4-heparin monoclonal antibody, purified from hybridoma supernatants (2). DNA 1 kb and 100 bp ladders were from New England BioLabs. GeneRuler high range DNA ladder (>50 kb; SM1351) was from ThermoFisher Scientific.

Human neutrophil isolation

Human whole blood collected into 3.8% sodium citrate (1:10 v/v) was layered over an equal volume of Lympholyte®-Poly density gradient (CedarLane Labs CL5071) and centrifuged at 500 g for 35 minutes at RT. The neutrophil layer was collected and washed with Hank's Balanced Salt Solution (HBSS, Gibco) by centrifugation at 350 g for 10 minutes. The pellet was resuspended in 3 mL ACK lysis buffer (Quality Biological), followed by immediate addition of 7 mL HBSS to dilute the leukocyte suspension. After centrifugation at 350 g for 5 more minutes, the pellet was resuspended in HBSS containing $CaCl_2$ and $MgCl_2$ (Gibco).

Intravenous DNA administration into mice

Wild-type (WT) C57BL/6 mice (Jackson Laboratory; strain #000664) or littermates deficient in murine PF4 (*CXCL4*$^{-/-}$) mice were given a single bolus of HMW DNA (100 µg/mouse) via tail vein prior to blood collection from the retro-orbital sinus 30 minutes post-DNA infusion into 1 mM EDTA. Mice were euthanized 4 hours post-DNA infusion, and blood was collected from the inferior vena cava into EDTA. Platelet-poor plasma was isolated by centrifugation at 2000 g for 20 minutes at RT for study.

Cystatin C ELISA

Clear-bottom MaxiSorp™ 96-well plate (442404, NUNC) was coated with cystatin C capture antibody (R&D Systems; DY1238) overnight at 4°C and blocked with 1% BSA for 1 hour at RT. Citrated plasma was diluted 1000-fold in 1% BSA, and incubated for 2 hours at RT in coated 96-well plate, followed by incubation with cystatin C detection antibody for 2 hours at RT. The

microplate was then incubated with streptavidin-peroxidase conjugate for 20 minutes at RT, and chromogenic substrate was then added for 2 minutes at RT. Reactions were stopped and absorbance was immediately measured at 450nm.

SUPPLEMENTAL FIGURES

Figure S1. Size separation of HMW and LWM NETs and DNA.

Representative images of DNA size separation. (**A**) 0.4% agarose gel size-separation of 10 µg HMW DNA and NETs with M = 50 kb size marker. (**B**) HMW NETs and DNA were digested with DNase I and size-separated on a 0.9% agarose gel with M (left) = 1 kb size marker and M (right) = 100 bp size marker. (**C**) Same as in (B) but following digestion with the indicated restriction enzymes. Af2 = AflII, BsrGI-HF = BG1 and Al1 = AluI.

Figure S2. LMW DNA-induced fibrin generation in PNP in the presence of PF4 and KKO.

(**A**) Lag time for LMW DNA-induced fibrin generation in the presence of increasing PF4 concentrations and 10 µg/mL KKO. (**B**) Lag time for LMW DNA-induced fibrin generation in the presence of low dose PF4 (1 µg/mL) and the indicated KKO concentrations. Data are mean ± SEM of 3 independent experiments.

Figure S3. LMW NETs- and DNA-induced thrombin generation in normal plasma, and fibrin formation in depleted plasma.

(**A**) Representative kinetic curves of thrombin generation over time, initiated by LMW NETs (left) and DNA (right) in the absence or presence of PF4 and KKO. **(B**) Representative kinetic curves of fibrin generation in PNP or FXI- or FXII-depleted plasma in the absence or presence of LMW DNA with or without added PF4 (20 µg/mL).

Figure S4. Effects of PF4 in LMW DNA-induced fibrin generation in depleted (dep) plasma supplemented with FXI and FXII.

(**A**) Lag time of LMW DNA-induced fibrin generation and effects of PF4 in FXI-dep plasma supplemented with indicated FXI concentrations. (**B**) Lag time of LMW DNA-induced fibrin generation and effects of PF4 in FXII-dep plasma supplemented with various FXII concentrations. Data are mean ± SEM of 3 independent experiments.

Figure S5. PF4 protects endothelial cells from LMW dsDNA and ssDNA-induced VWF release mediated by TLR9.

(**A** and **B**) Representative images of HUVECs showing immunofluorescent staining for VWF (red) and nuclei (blue) upon exposure to LMW dsDNA (**A**) or ssDNA (**B**) fragments in the absence or presence of PF4. Mean fluorescence intensity (MFI) of released VWF from HUVECs exposed to

LMW dsDNA (**C**) or ssDNA (**D**), with or without the TLR9 inhibitor HCQ. Data were normalized to MFI of untreated cells without exposure to dsDNA or inhibitor (as indicated by dotted lines). Exposure to α-thrombin (C) or TNFα (D) serves as a positive control. Data are mean ± SEM of at least 3 independent experiments.

Figure S1

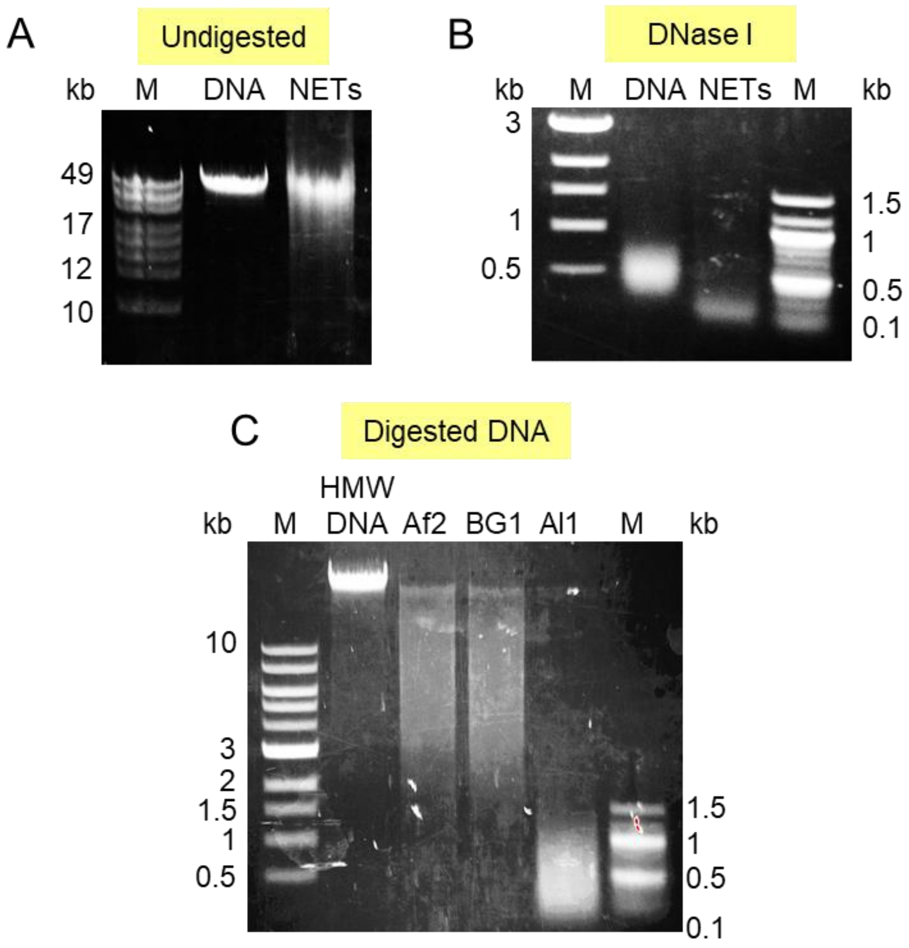
A
Undigested
kb
M
DNA
NETs
49
17
12
10
B
DNase I
kb
M
DNA
NETs
M
kb
3
1
0.5
1.5
1
0.5
0.1
C
Digested DNA
HMW
kb
M
DNA
Af2
BG1
Al1
M
kb
10
3
2
1.5
1
0.5
1.5
1
0.5
0.1

Figure S2

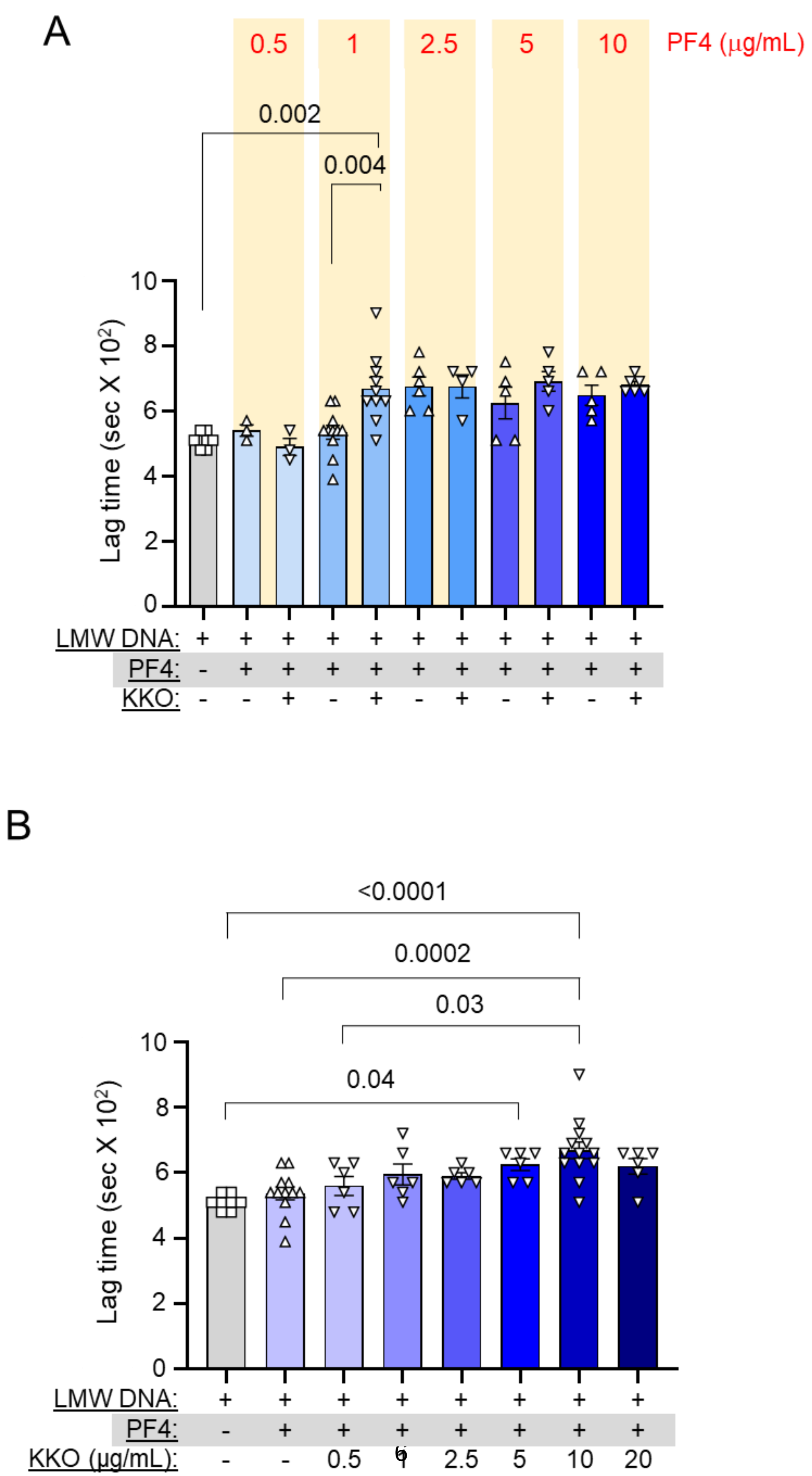

A
0.5
1
2.5
5
10
PF4 (μg/mL)
0.002
0.004
Lag time (sec X 10²)
LMW DNA: + + + + + + + + + + +
PF4: - + + + + + + + + + +
KKO: - - + - + - + - + - +
B
<0.0001
0.0002
0.03
0.04
Lag time (sec X 10²)
LMW DNA: + + + + + + + +
PF4: - + + + + + + +
KKO (μg/mL): - - 0.5 1 2.5 5 10 20

Figure S3

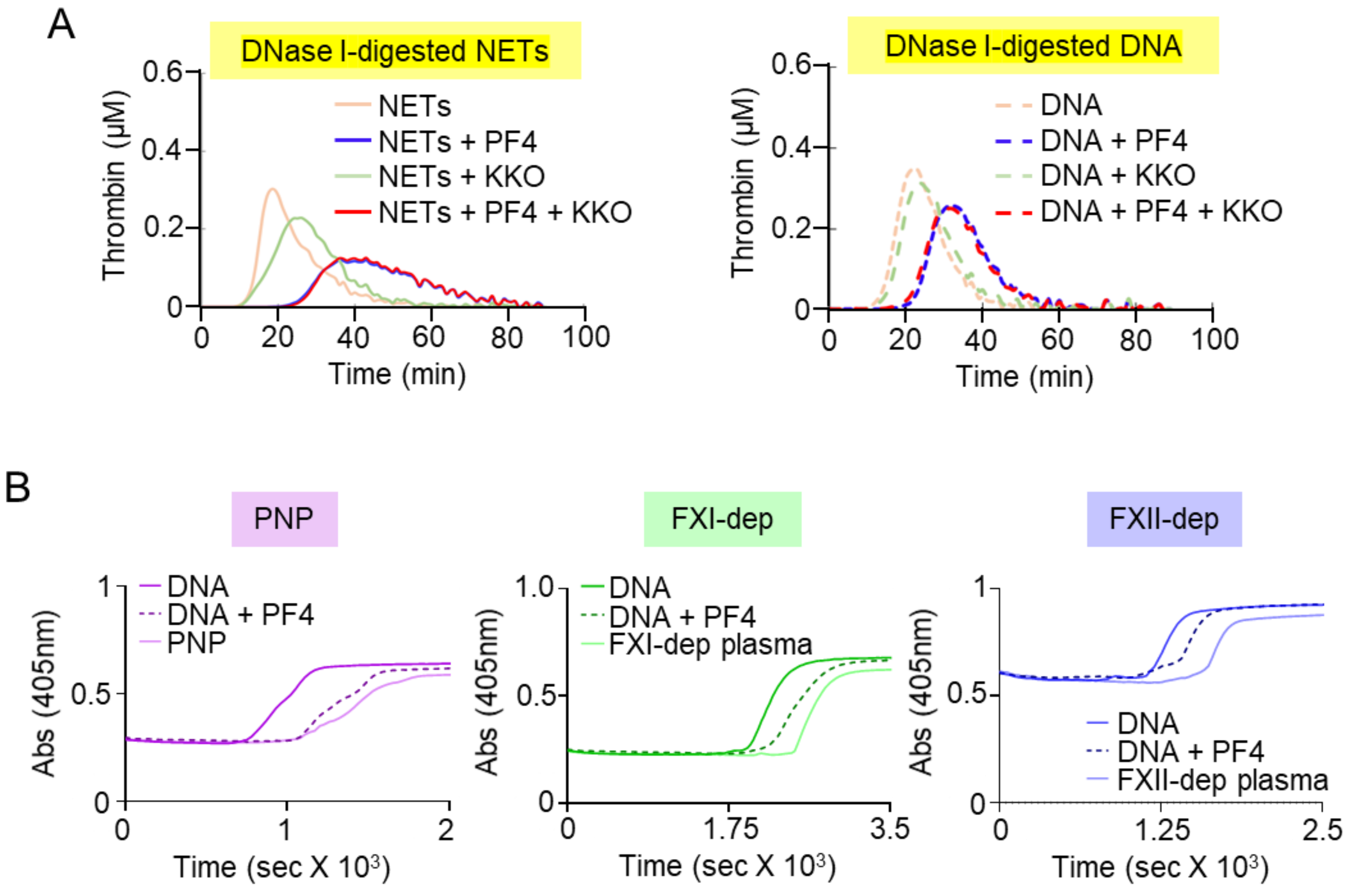
A
DNase I-digested NETs
Thrombin (µM)
0.6
0.4
0.2
0
NETs
NETs + PF4
NETs + KKO
NETs + PF4 + KKO
0
20
40
60
80
100
Time (min)
DNase I-digested DNA
Thrombin (µM)
DNA
DNA + PF4
DNA + KKO
DNA + PF4 + KKO
Time (min)
B
PNP
Abs (405nm)
1
0.5
0
DNA
DNA + PF4
PNP
Time (sec X 10³)
FXI-dep
1.0
DNA
DNA + PF4
FXI-dep plasma
1.75
3.5
FXII-dep
DNA
DNA + PF4
FXII-dep plasma
1.25
2.5

Figure S4

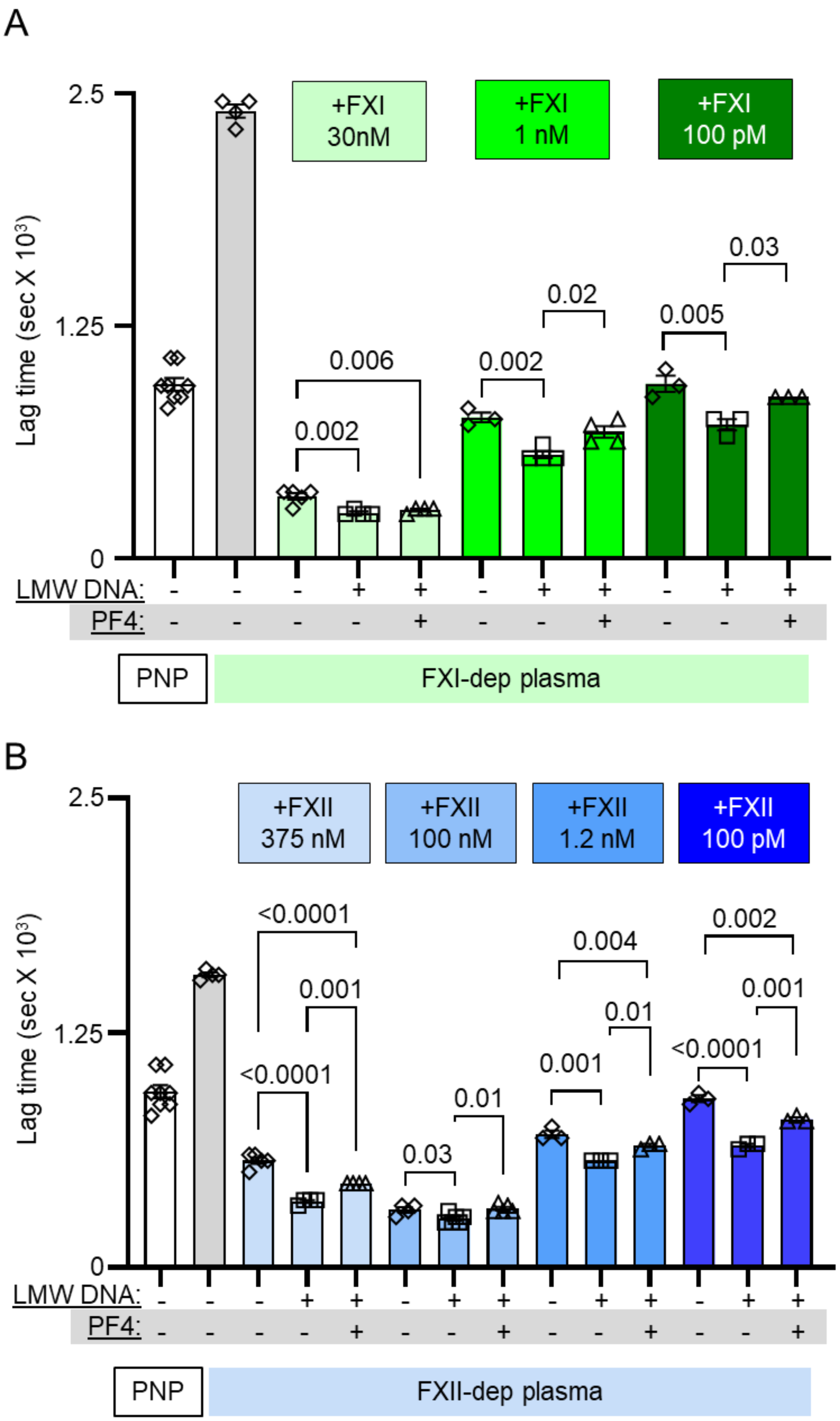
A
Lag time (sec X 10³)
2.5
1.25
0
+FXI
30nM
+FXI
1 nM
+FXI
100 pM
0.006
0.002
0.002
0.02
0.005
0.03
LMW DNA: - - - + + - + + - + +
PF4: - - - - + - - + - - +
PNP
FXI-dep plasma
B
Lag time (sec X 10³)
2.5
1.25
0
+FXII
375 nM
+FXII
100 nM
+FXII
1.2 nM
+FXII
100 pM
<0.0001
0.001
<0.0001
0.03
0.01
0.004
0.01
0.001
0.002
0.001
<0.0001
LMW DNA: - - - + + - + + - + + - + +
PF4: - - - - + - - + - - + - - +
PNP
FXII-dep plasma

Figure S5

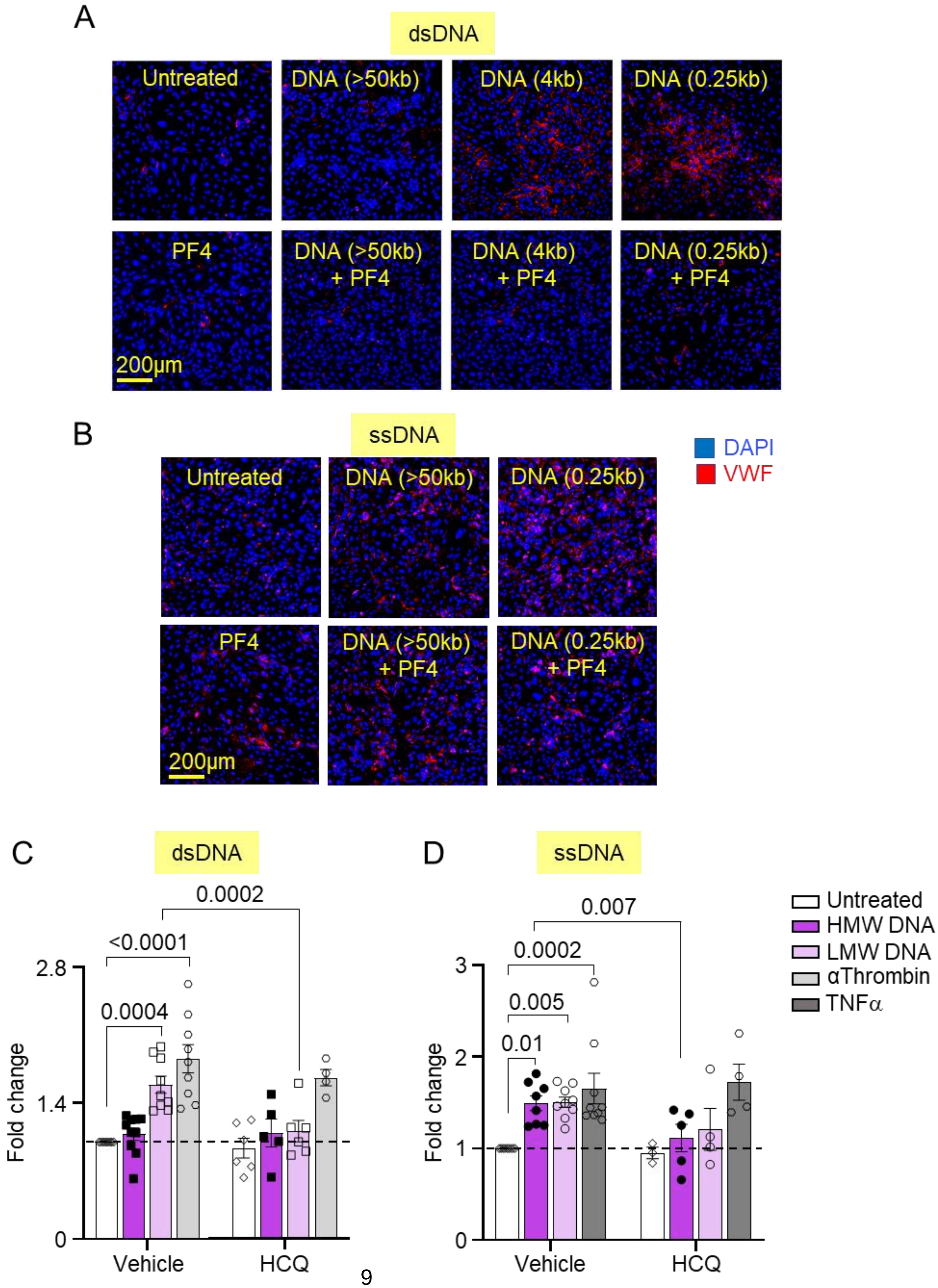
A
dsDNA
Untreated
DNA (>50kb)
DNA (4kb)
DNA (0.25kb)
PF4
DNA (>50kb) + PF4
DNA (4kb) + PF4
DNA (0.25kb) + PF4
200µm
B
ssDNA
DAPI
VWF
Untreated
DNA (>50kb)
DNA (0.25kb)
PF4
DNA (>50kb) + PF4
DNA (0.25kb) + PF4
200µm
C
dsDNA
0.0002
<0.0001
0.0004
Fold change
2.8
1.4
0
Vehicle
HCQ
D
ssDNA
0.007
0.0002
0.005
0.01
Fold change
3
2
1
0
Vehicle
HCQ
Untreated
HMW DNA
LMW DNA
αThrombin
TNFα

REFERENCES

1. Cuker A, Rux AH, Hinds JL, et al. Novel diagnostic assays for heparin-induced thrombocytopenia. *Blood.* 2013;121(18):3727-3732.
2. Arepally GM, Kamei S, Park KS, et al. Characterization of a murine monoclonal antibody that mimics heparin-induced thrombocytopenia antibodies. *Blood.* 2000;95(5):1533-1540.
